# Novel Cost-Effective Model for Training Post-intubation Endotracheal Tube Placement Confirmation

**DOI:** 10.7759/cureus.57830

**Published:** 2024-04-08

**Authors:** Kristine Jeffers, Brandon Murdock, Steven Siemieniak, Melissa Myers

**Affiliations:** 1 Emergency Medicine, Brooke Army Medical Center, Fort Sam Houston, USA

**Keywords:** point-of-care ultrasound, airway procedures, post intubation, training model, endotracheal intubation

## Abstract

Intubation in emergency settings demands rapid confirmation of endotracheal tube (ETT) placement for establishing a definitive airway. Current methods, including capnography and auscultation, have limitations. This study introduces a cost-effective and easily accessible training model for confirming ETT placement using ultrasound, aiming to improve training and patient outcomes. We developed a gelatin and psyllium-based model that simulates adult ETT intubation, offering an alternative to costly cadaveric models. The model's construction is described, with materials costing approximately $7.34 per unit. Preliminary results show promise in simulating tracheal and esophageal intubation scenarios. This novel model provides an ethical and economical solution for training healthcare professionals in the ultrasound confirmation of ETT placement, paving the way for further validation and adoption in medical education.

## Introduction

Intubation is a commonly performed procedure in the emergency department that requires rapid confirmation after endotracheal tube (ETT) insertion to establish a definitive airway. The gold standard is visualizing passage of the ETT through the glottis. However, there are a number of other methods to confirm proper ETT placement. These include colorimetric capnography, auscultation of bilateral lung sounds, and continuous end-tidal capnography [1]. While simple to use, these methods require adequate ventilation and/or adequate perfusion which can be challenging in cardiac arrest [1]. Continuous end-tidal capnography is not always readily available. Even if available, it is only 93% sensitive and can decrease to 72% sensitive in cardiac arrest patients [2,3]. Ultrasound for ETT placement confirmation was first published in 1987, suggesting its potential as an effective adjunct. Early use of ultrasound may also reduce the number of x-rays performed for ETT confirmation, sparing patients from radiation exposure [4]. As ultrasound becomes more available and accessible at the bedside, confirmation of proper ETT placement with point-of-care ultrasound (POCUS) shows promise for improved patient outcomes.

Ultrasound confirmation of ETT insertion into the trachea at the bedside is a rapid and effective method for verifying correct placement. Transtracheal ultrasound to confirm ETT placement relies on the differences in anatomy between the trachea and the esophagus. The trachea has cartilaginous rings and the esophagus is a relatively flat and compressed structure. If the ETT is inserted into the esophagus it will becomes readily visible as two hyperechoic curvilinear structures with posterior shadowing and reverberation artifact known as the double tract sign [5]. A systematic review performed by Peksa et al. demonstrates that ultrasound is 98.7% sensitive and 97.1 % specific, with mean time to confirmation at 13.0 seconds [6]. Their review demonstrated that transtracheal sonography is “rapid to perform, with an acceptable degree of sensitivity and specificity for the confirmation of endotracheal intubation…and should be considered when quantitative capnography is unavailable or unreliable” [6]. While transtracheal confirmation is becoming more accessible and has promise as an adjunct to confirmation, it requires proper training to attain proficiency.

Most studies on this topic rely on cadaveric data as a model for ETT placement confirmation, which is costly and resource intensive. There has been a model proposed using a block of beef gelatin and psyllium fiber creating two cored tubes with a 10mL syringe [7]. With the core fully inserted, it creates the simulation of a tracheal intubation and when partially inserted it simulates an esophageal intubation. When these models were used in training, they improved image recognition and time to image acquisition on intubated NICU patients [7]. While this data is promising, there has not been a novel model produced to simulate adult ETT intubation. For this, we have developed a novel training model to simulate the proportions and image quality of adult ETT intubation. Our study goal is to demonstrate that our model is non-inferior for intubation confirmation using ultrasound compared to training with cadaveric models as this is the most common model for training. If this is the case, we will be able to create a low cost and high-fidelity method for junior residents and learners to practice ETT confirmation with ultrasound.

## Technical report

**Figure 1:**
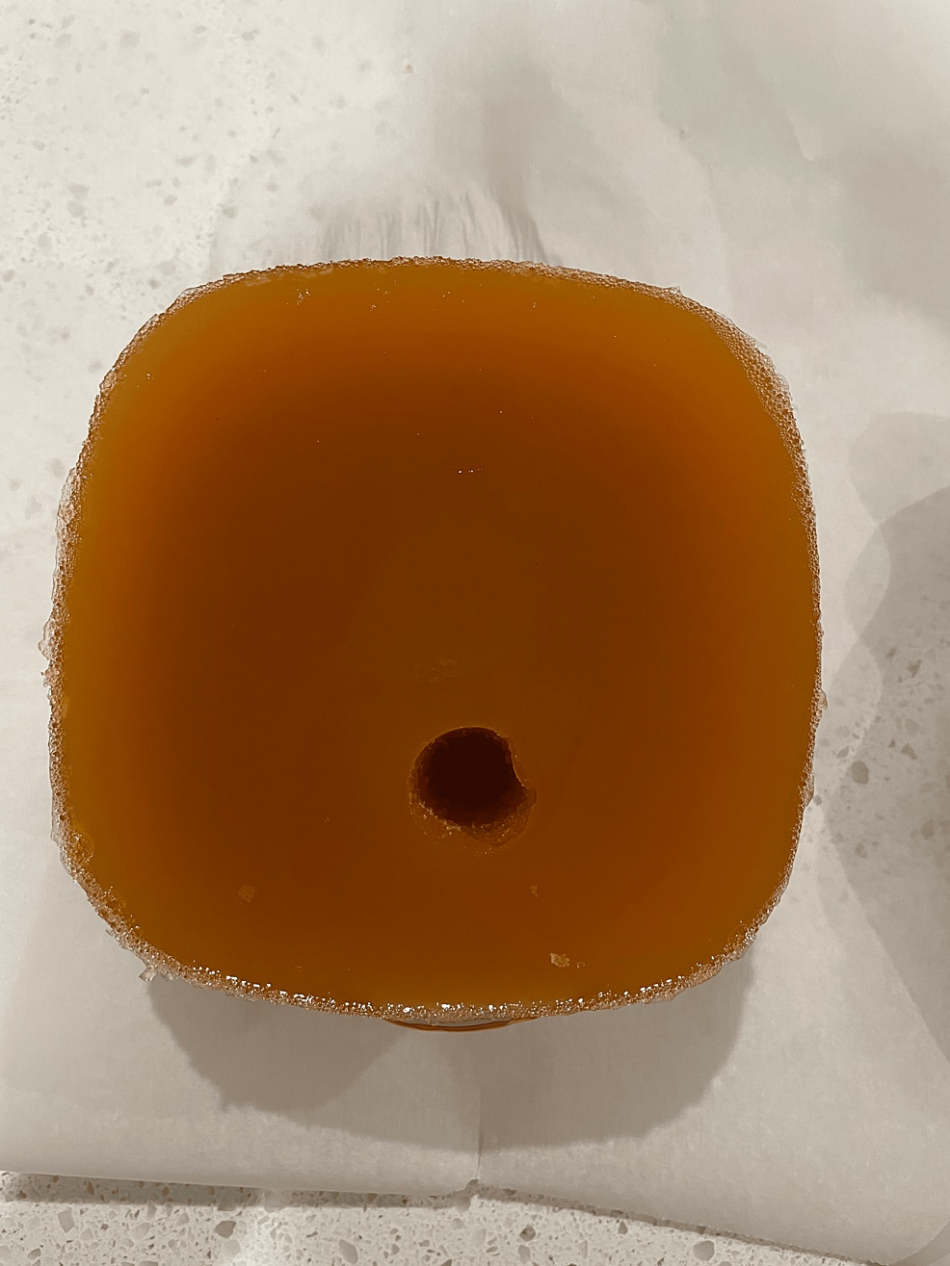
Beef gelatin model with single lumen consistent with endotracheal intubation

**Figure 2:**
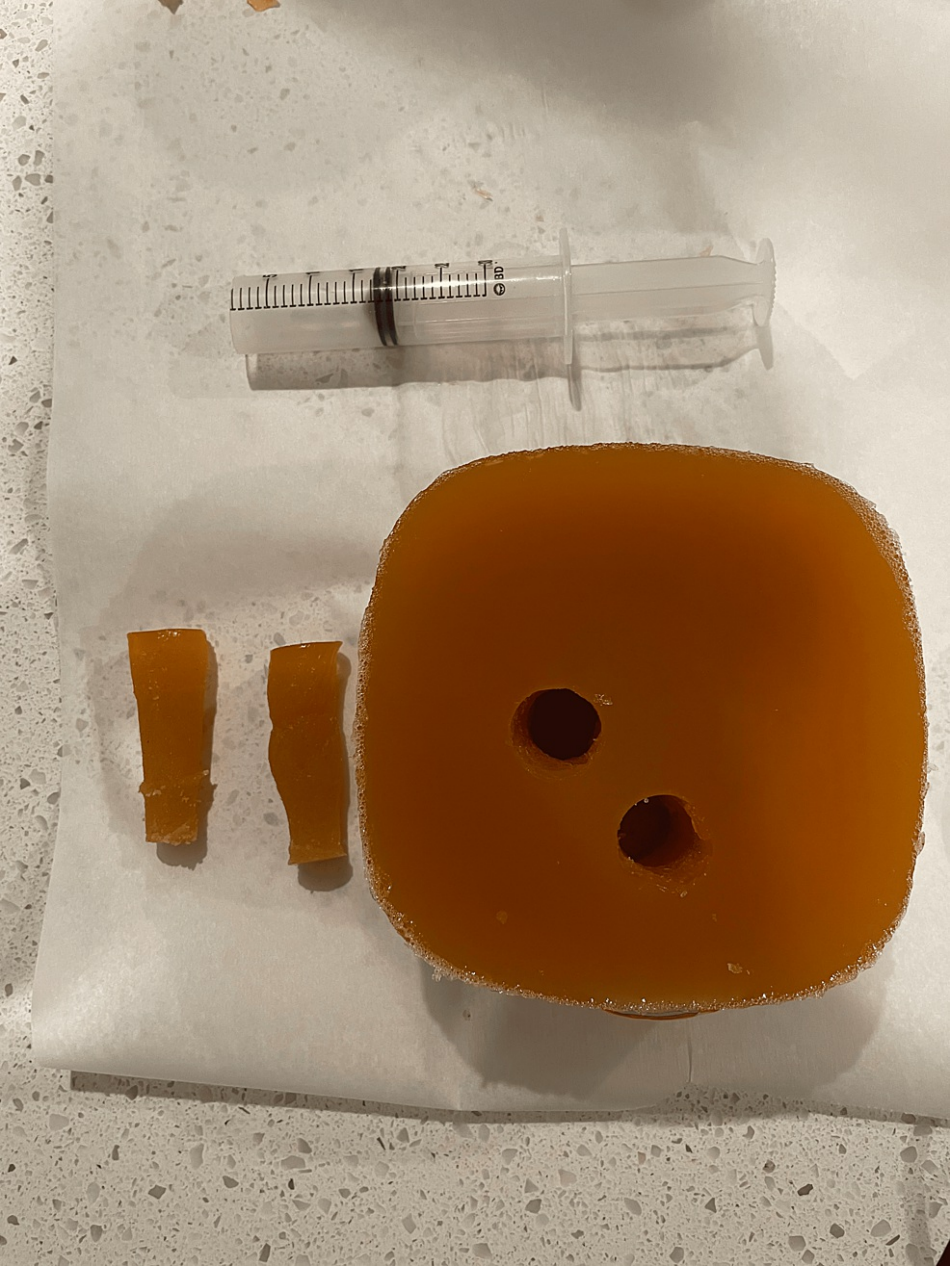
Beef gelatin model with two lumens consistent with esophageal intubation with cutoff 30 cc syringe and gelatin cores used to make the model

**Figure 3:**
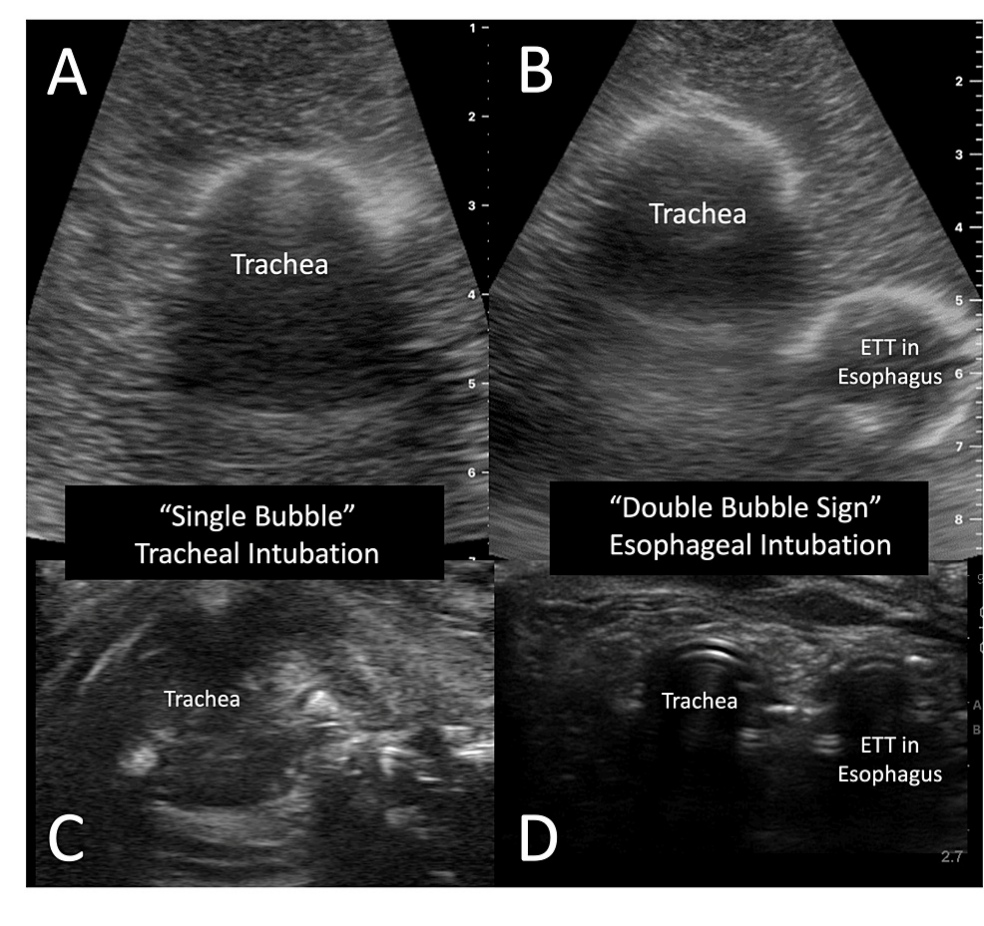
(A) Model endotracheal intubation; (B) Model esophageal intubation; (C) Cadaver endotrachal intubation; (D) Cadaver esophageal intubation

In our attempt to make a low-cost model, we used materials commonly found around the emergency department or common household items. For the mold a plastic Tupperware container holding 1 L was used, but any square container will suffice. The gelatin model was made using a mixture of four cups of boiling water, 41 g of psyllium to 84 g of gelatin [8]. Once the water was brought to a boil, the gelatin was slowly mixed in while stirring to prevent clumping. After the gelatin was dissolved, the psyllium was added slowly while mixing thoroughly. The model was then placed in a household refrigerator at 4°C overnight or for at least six hours. Once solidified, the mold was removed from the plastic container, and using a 30cc syringe with the end cut off, two lumens were made in the mold. The first lumen, representing the trachea, was placed in the center and approximately 3 cm deep from the anterior edge (Figure 1). The second lumen, representing the esophagus, was placed 1 cm deeper and 1 cm to the right of the tracheal lumen (Figure 2). The second core from the hole, made to represent the esophagus, was saved to allow it to be either removed or replaced back into the model. When simulating appropriate endotracheal intubation, the core is added to the model versus when simulating endotracheal intubation the core is removed. Model images were obtained using a Butterfly IQ ultrasound probe on the musculoskeletal preset for comparison with images obtained from cadaveric models (Figure 3). The model was then replaced back in the container and placed in the refrigerator to maintain its integrity and prolong its use. The model was used by greater than 25 users for teaching ultrasound for confirmation of endotracheal intubation and lasted for three weeks in the refrigerator prior to developing mold. 

## Discussion

POCUS has expanded its use and application in the emergency department into numerous areas of care. While the use and application of this resource has expanded, there are limited tools for teaching these new techniques. There are some commercially available training models, but there are no ready-made resources for teaching confirmation of ETT intubation using ultrasound. There is also an ethical dilemma as the most realistic training models are cadaver based. While similar anatomically, cadaveric models are expensive and resource intensive. We proposed a model made of psyllium and gelatin to learn the difference of endotracheal and esophageal intubation. The cost of the supplies to make this model was approximately $7.34 (Table 1) and takes approximately one hour to make and six hours to chill, which allows for a relatively inexpensive and simple training model.

**Table 1:** Cost breakdown of the model and components

Item	Cost
Container	$2.48 per container
Gelatin	$0.045/g x 84 g = $3.78
Psyllium	$0.018/g x 41 g= $0.74
30 cc syringe	$0.34 per syringe
Total	$7.34

While gelatin was used for this model because of the ease of access and the reduction in cost, there are some models that use commercially available ballistic gelatin. Because gelatin was used for the model, this only lasts for a couple weeks at a time and up to two months if refrigerated before losing integrity [9]. Psyllium was added as a filler to better represent the subcutaneous tissue that is seen in cadaveric and live tissue models (Figure 3). 30 cc syringes are easily accessible in the emergency department and most closely match the average size of an adult male trachea, which can range from 15 to 25 mm [10]. The syringe's outer diameter measures 25.27 mm, making it the most similar in size. Using this model, either endotracheal intubation or esophageal intubation can be simulated by making one or two tracts with the syringe. Compared to cadaveric images, the gelatin and psyllium model similarly display one or two tracts to teach learners about the double-tract sign to differentiate endotracheal versus esophageal intubation.

We describe a novel model that can provide reliable training to learners for ultrasound confirmation of ETT placement, but the model requires further validation and comparison to the current training standard of cadaveric models. More studies are required to further evaluate to validate the use of this model for training.

## Conclusions

Use of ultrasound to confirm ETT placement can avoid a catastrophic outcome in the case of esophageal placement. In addition, ultrasound offers an alternative to end tidal capnography in resource limited settings. We propose a novel and cost-effective model for post-intubation ETT confirmation using a psyllium and gelatin mixture. The proposed model, designed to simulate adult intubation proportions and image quality, offers a low-cost alternative to traditional cadaveric models. This model provides an alternative to the resource-intensive nature of existing training methods. This model, despite its affordability, maintains high fidelity in training for ultrasound confirmation and holds promise for enhancing the skills of junior residents and learners. Further studies are needed to establish the efficacy and reliability of this novel training model compared to the current cadaveric standard.
